# Hippocampal involvement in nonpathological déjà vu: Subfield vulnerability rather than temporal lobe epilepsy equivalent

**DOI:** 10.1002/brb3.996

**Published:** 2018-06-05

**Authors:** Eva Pešlová, Radek Mareček, Daniel J. Shaw, Tomáš Kašpárek, Martin Pail, Milan Brázdil

**Affiliations:** ^1^ Department of Neurology Brno Epilepsy Center St. Anne’s University Hospital and Medical Faculty of Masaryk University Brno Czech Republic; ^2^ Multi‐modal and Functional Neuroimaging Research Group CEITEC ‐ Central European Institute of Technology Masaryk University Brno Czech Republic; ^3^ Behavioral and Social Neuroscience Research Group CEITEC ‐ Central European Institute of Technology Masaryk University Brno Czech Republic; ^4^ Department of Psychiatry Faculty Hospital Brno and Medical Faculty of Masaryk University Brno Czech Republic

**Keywords:** deja vu, hippocampal subfields, hippocampal vulnerability, mesial temporal lobe epilepsy, schizophrenia

## Abstract

**Introduction:**

Morphological correlates of nonpathological déjà vu (DV) have been identified recently within the human brain. Significantly reduced gray matter volume (GMV) within a set of cortical and subcortical regions reported in subjects experiencing DV seems to mirror the distribution of GMV reduction in mesial temporal lobe epilepsy (MTLE) patients but vary in terms of the hippocampus. Another condition associated with hippocampal GMV reduction and DV alike disturbance in memory processing is schizophrenia (SCH). Here, we tested the hypothesis that hippocampal involvement in nonpathological DV resembles more closely the pattern of GMV decrease observed in MTLE compared with that occurring in SCH.

**Methods:**

Using automated segmentation of the MRI data we compared the medians of GMV within 12 specific hippocampal subfields in healthy individuals that do (DV+; *N* = 87) and do not report déjà vu experience (DV−; *N* = 26), and patients with MTLE (*N* = 47) and SCH (*N* = 29). By Pearson correlation, we then evaluated the similarity of MTLE and SCH groups to DV+ group with respect to spatial distribution of GMV deviation from DV− group.

**Results:**

Significant GMV decrease was found in MTLE group in most of the subfields. There were just trends in the hippocampal GMV decrease found in DV+ or SCH groups. Concerning the spatial distribution of GMV decrease, we revealed statistically significant correlation for the left hippocampus for SCH vs DV+. Otherwise there was no statistically significant correlation.

**Conclusions:**

Our findings reveal structural features of hippocampal involvement in nonpathological DV, MTLE, and SCH. Despite our expectations, the pattern of GMV reduction in the DV+ relative to the DV− group does not resemble the pattern observed in MTLE any more than that observed in SCH. The highly similar patterns of the three clinical groups rather suggest an increased vulnerability of certain hippocampal subfields; namely, Cornu Ammonis (CA)4, CA3, dentate gyrus granular cell layer (GC‐DG), hippocampal–amygdaloid transition area (HATA) and subiculum.

## 1 INTRODUCTION

Déjà vu (DV) is a complex phenomenon of erroneous familiarity. When experienced by up to 76% of the healthy population (Adachi et al., 2003), DV is believed to reflect aberrant yet nonpathological brain function among memory‐related neural systems (Shaw, Mareček, & Brázdil, 2015). On the other hand, DV experience occurs also as a manifestation of neurological diseases, such as temporal lobe epilepsy (as a type of aura; Illman, Butler, Souchay, & Moulin, 2012), and in several psychiatric disorders, such as anxiety or depression (Richardson & Winokur, 1967). While different in frequency and duration, DV appears to be qualitatively same whether it occurs as a benign nonpathological event or in the context of epilepsy (Warren‐Gash & Zeman, 2014).

Recently, morphological correlates of nonpathological DV have been identified within the human brain using magnetic resonance imaging (MRI, Brázdil et al., 2012; Labate et al., 2015). Using highly sensitive source‐based morphometry to investigate a large cohort of healthy subjects, significantly reduced gray matter volume (GMV) was revealed within bilateral mesiotemporal regions (namely hippocampi and parahippocampal gyri), insular cortices, superior temporal sulci, basal ganglia and thalami in subjects reporting prior experience of DV compared to those reporting no such experience. Importantly, GMV within these regions correlated inversely with the frequency of DV experience (Brázdil et al., 2012).

This finding converges closely with the results of a study by Shaw et al. (2015), whereby GMV reductions among medial temporal lobe structures were found to covary more strongly in healthy individuals reporting a higher frequency of DV experience (Shaw et al., 2015).

More direct evidence for the role of medial temporal lobes in DV experience comes from the electrophysiological study by Bancaud, Brunet‐Bourgin, Chauvel, and Halgren (1994). These authors applied intracerebral electrical stimulation to evoke DV in patients with temporal lobe epilepsy. They concluded that the hippocampus and amygdala play key roles in the genesis of DV experience, with the temporal neocortex providing a secondary but important contribution (Bancaud et al., 1994).

The hippocampal formation is a gray matter region essential in memory functions. It consists of anatomically and functionally distinct subfields: presubiculum, parasubiculum, subiculum, Cornu Ammonis (CA)1, CA2, CA3, CA4, granular cell layer of the dentate gyrus (GC‐DG), fimbria, hippocampal–amygdaloid transition area (HATA), hippocampal molecular layer (molecular layer HP), fissure and tail. Some authors include also entorhinal cortex which serves as the main “interface” between the hippocampus and other parts of the brain (Andersen, Morris, Amaral, Bliss, & OKeefe, 2006). The trisynaptic pathway from the entorhinal cortex, inducing serial excitatory transmission through CA4, GC‐DG, CA2‐3, and CA1, and leading back to the entorhinal cortex through the subiculum, is then considered to be a fundamental network involved in learning and memory (Tamminga, Stan, & Wagner, 2010).

Aside from DV phenomenon, hippocampal volume is decreased frequently in the setting of epilepsy. The set of brain regions distinguishing brain experiencing DV (Brázdil et al., 2012) remarkably mirrored the distribution of GMV reduction in subjects with mesial temporal lobe epilepsy (MTLE) with hippocampal sclerosis (HS) involving hippocampal and parahippocampal regions, entorhinal and perirhinal cortices, amygdala, lateral temporal neocortex, thalamic and striatal nuclei, cingulate gyrus, insula, and cerebellum (Brázdil et al., 2009; Keller & Roberts, 2008; Labate et al., 2015; Pail, Brázdil, Mareček, & Mikl, 2010). Interestingly the pattern of GMV reduction seems to be distinctive within the hippocampi (Brázdil & Zeman, 2013).

A significant reduction in hippocampal volume was also found in several mental disorders, such as in the case of schizophrenia, where both neurodevelopmental and degenerative processes are thought to contribute to the pattern of neuroanatomic changes (Haukvik et al., 2015; Honea, Crow, Passingham, & Mackay, 2005; Kühn et al., 2012; Mathew et al., 2014). Hippocampus is presumably a key structure in pathophysiology of schizophrenia (Lodge & Grace, 2011). Impaired hippocampal recruitment during the memory recall (Heckers et al., 1998; Weiss et al., 2003) combined with attribution of strong behavioral importance to stimuli that would be otherwise safely ignored (termed “aberrant salience”) is suggested to potentially underlie delusional or hallucinatory states in schizophrenia (Kapur, 2003). Remarkably, this concept of providing irrelevant context to currently processed percepts/cognitions corresponds with one of the widely accepted theories considering mechanisms behind déjà vu. It is believed that aberrant memory processing leads to implicit familiarity of unrecognized stimuli (Brown, 2003).

The aforementioned results of morphology studies in healthy individuals and electrophysiological investigations with epileptic patients point to the important role of the hippocampal formation in the genesis of pathological and nonpathological DV.

The question is whether the morphological correlate of nonpathological DV in terms of hippocampal subfields converges with this one of MTLE as a condition with corresponding morphological correlate in various sites of the brain or with the schizophrenia which carries similar concept considering underlying mechanisms.

To answer this inquiry and to define the precise involvement of the hippocampus in DV, we used automated segmentation to assess and compare GMV across the hippocampal subfields in four patient groups. The first group consisted of healthy individuals reporting DV experience regarded as nonpathological (DV+), the second group consisting of those reporting no such experience (DV−), and the third and fourth group comprising patients suffering from MTLE and schizophrenia (SCH) (with their DV considered as pathological).

Based on reports of high neuroanatomical similarity in healthy individuals reporting nonpathological DV and MTLE patients in whom pathological DV occurs frequently, we hypothesized that patterns of GMV reduction across hippocampal subfields in the DV+ group would resemble those measured in MTLE but differ from those observed in the SCH group with a more complex pattern of clinical presentation and distinct underlying hippocampal neuropathology.

## 2 METHODS

### 2.1 Subjects

Our sample comprised a total of 189 subjects participated in the study.

The MTLE group consisted of a left‐sided (L‐MTLE; *N* = 27; 8M, 19F; median age = 40.2 years; range = 17.7 – 54.6 years) and right‐sided subgroup (R‐MTLE; *N* = 20; 6M, 14F; median age = 38.2 years; range = 24.8 – 61.1 years). For the L‐MTLE subgroup, the median age at the time of disease onset was 3 years (range 1 – 35 years), the median disease duration was 32.5 years (range 6 – 54 years), and the median seizure frequency was 4 seizures per month (range 2 – 135 seizures). For the R‐MTLE subgroup, the median age at the time of the disease onset was 5.5 years (range 8 months – 40 years), the median disease duration was 29.5 years (range 8 – 55 years), and the median seizure frequency was 4.75 seizures per month (1 – 13).

At the time of testing, the subgroups did not differ significantly in age (*p* = .97, *T*–test), gender (*p* = .48; *T*–test), disease duration (*p* = .60, *T*–test) or seizure frequency (*p* = .40, *T*–test). There was no significant variance between the subgroups in their positive history of febrile convulsions (seven in L‐MTLE and six in R‐MTLE; *p* = .74, *T*–test). Majority of the subjects had a history of complex partial seizures. Ten patients in the L‐MTLE subgroup and eight patients in the R‐MTLE subgroup also presented secondary generalized seizures. One patient with L‐MTLE and one with R‐MTLE had simple partial seizures. DV in the context of aura was reported solely by one patient. The only statistically significant divergence between the subgroups was in the number of prescribed antiepileptic drugs (number of types): a mean of 2.3 medications for the L‐MTLE subgroup, and a mean of 1.8 medications for R‐MTLE subgroup (*p* = .03, *T*‐test). The diagnosis of unilateral MTLE was based on a consonance of history data, ictal and interictal EEG findings (long‐term semi‐invasive video‐EEG monitoring with sphenoidal electrodes), ictal semiology, neuropsychology, neuroimaging findings, and fulfillment of the diagnostic criteria for MTLE according to the International League Against Epilepsy (ILAE) criteria (Commission on Classification and Terminology of ILAE, 1989). As another inclusion criteria was set also hippocampal sclerosis, which is a substrate characterizing classical TLE (Hogan, 2001). Unilateral hippocampal sclerosis MRI evidence concordant with the EEG lateralization of the epileptogenic zone was found in all subjects. None of the subjects revealed any other structural brain lesions on MRI scans, and none had undergone previous intracranial surgery. All subjects had been seizure free for 24 hr prior to the MRI investigation.

The SCH group comprised of 29 subjects (23M, 6F; median age = 33 years; range = 19.3 – 45.1 years) with a median disease duration of 4.5 years (range = 1 ‐ 288 months). The symptom severity in these patients was assessed with Positive and Negative Syndrome Scale (PANSS; Kay, Fiszbein, & Opler, 1987). Patients scored a median of 14 points (range = 7 – 20 points) on the positive scale, a median score of 21 points (range = 10 – 29 points) on the negative scale, and a median of 38 points (range = 21 – 53 points) on the general psychopathology scale. The median of experienced episodes was 2 (range = 1 ‐ 8 episodes) during the course of the disease, and all subjects were on stable antipsychotic medication with median of 1 (range = 1‐2 drugs, all atypical antipsychotics). None of the subjects reported history of epilepsy or signs of hippocampal sclerosis on MRI.

The DV+ group comprised of 87 healthy volunteers (45M, 42F; median age = 24.1 years; range = 19.1 – 46.2 years) with no personal or family history of epilepsy or schizophrenia. All subjects completed the Inventory for Déjà vu Experiences Assessment (IDEA)—a questionnaire used widely in previous DV research (Sno, Schalken, de Jonghe, & Koeter, 1994)—and answered “yes” to the question A1:”Have you ever had the feeling of having experienced a sensation or situation before in exactly the same way when in fact you are experiencing it for the first time?”.

The DV− group included 26 healthy volunteers (14M, 12F; median age = 24.2 years; range = 20.3 – 49.5 years) with no personal or family history of epilepsy or schizophrenia. These individuals answered “never” to question A1 of the IDEA questionnaire.

Group factor testing reached statistical significance in the case of age (Kruskal‐Wallis test H[4,189] = 59.6, *p* < .001) and gender (Pearson Chi‐square test, chi‐square = 17.9, *p* = .001).

Written informed consent was obtained from each participant after all the procedures were explained in full. The study received the approval of the local ethics committee.

### 2.2 MRI data acquisition

We acquired a T1 MPRAGE high resolution MRI for each subject with a Siemens Symphony 1.5T MR scanner (160 sagittal slices; matrix size 256 × 256, resampled to 512 × 512; TR = 1,700 ms; TE = 3.96 ms; FOV = 246 mm; FA = 15°; slice thickness = 1.17 mm; native in plane resolution = 0.96 mm). Eight images were discarded due to severe motion artifacts identified by visual inspection. The resulting images comprised the data described above and analyzed as follows.

### 2.3 MRI data preprocessing

We used the Freesurfer 6.0 toolbox for data preprocessing with additional tool for hippocampal subfields segmentation (recon‐all –all –hippocampal‐subfields‐T1 command) (Iglesias et al., 2015). The output of the preprocessing contained an information of the hippocampal subfields volumes, namely parasubiculum, presubiculum, subiculum, CA1, CA3, CA4, GC‐DG, HATA, fimbria, molecular layer HP, fissure and tail as well as an estimate of the total intracranial volume (TIV). The subfield volumes were corrected for age and gender effects using linear regression and for variability in head volume using normalization by TIV. We refer to these values as corrected subfield volumes (CSV).

### 2.4 Statistics

The purpose of the study was to map GMV within distinctive hippocampal subfields, and to perform comparisons in these measures of GMV among the DV+, DV−, SCH, and MTLE groups. We carried out two statistical tests with the STATISTICA 12 (StatSoft, Inc., USA).

#### 2.4.1 Subfield specific GMV decrease

To reveal subfield specific GMV change in DV+, SCH, and MTLE group relative to the DV− group, we performed Mann‐Whitney tests on CSV for each subfield and group (DV+, SCH and MTLE vs. DV−). The significance level was set to *p* < .05.

#### 2.4.2 Spatial distribution of GMV decrease

We computed median of the CSV volumes (mCSV) across subjects for each group and subfield. To identify which patient group resembles more the DV+ group considering the spatial pattern of GMV deviations from DV− we further computed the deviation of mCSV between DV+/MTLE/SCH and DV− (dmSCV). The dmCSV patterns were assessed using Pearson correlation coefficient, that is, we computed correlation coefficient based on dmSCV for DV+ vs MTLE in affected hippocampus and DV+ vs SCH in both‐sided hippocampi. The significance level of correlation coefficients was set to *p* < .05.

## 3 RESULTS

### 3.1 Subfield specific GMV decrease

**Figure 1:**
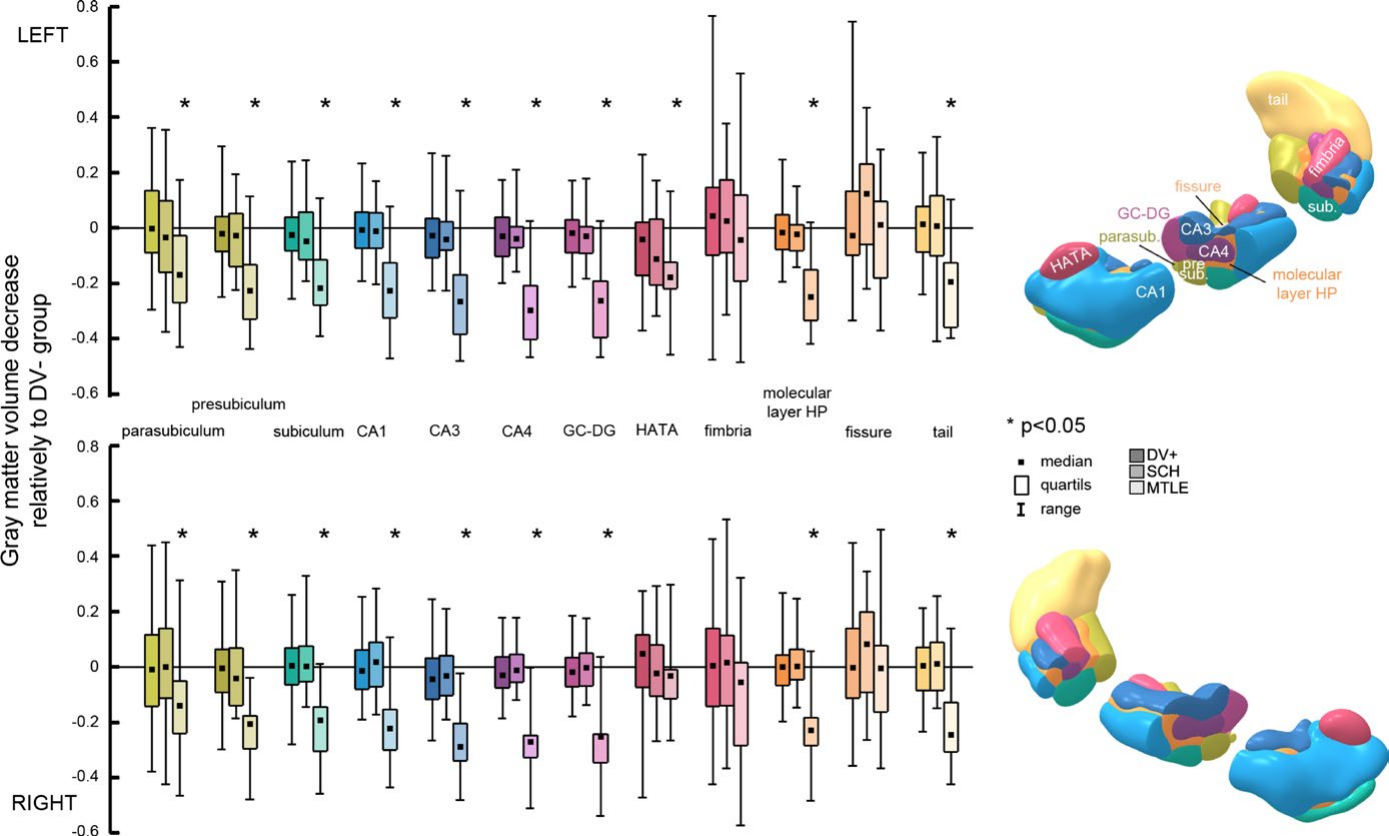
Relative gray matter volume (GMV) decrease in DV+, schizophrenia (SCH), and mesial temporal lobe epilepsy (MTLE) compared to DV− in distinctive hippocampal subfields. Asterisks mark subfields within which GMV change reached statistical significance (*p* < .05) after Bonferroni correction. The *Y*‐axis indicates extent of GMV change relative to the DV− group

Significant GMV decrease in the MTLE group relative to DV− was found in all hippocampal subfields with the exception of bilateral HATA, fimbria and fissure. There was no significant change of SCV found in DV+ or SCH groups. The extent and significance of the decrease shows Figure 1.

### 3.2 GMV change in Mesial temporal lobe epilepsy

In the MTLE group, only the ipsilateral hippocampus was measured regarding the distinctive manner of hippocampal involvement in the left and right‐sided MTLE (Pail et al., 2010).

Significant GMV decrease of the MTLE group relative to DV− was found in all hippocampal subfields with the exception of bilateral HATA, fimbria and fissure. The largest relative decrease was found in the CA4, CA3 and GC‐DG subfields. More specifically, on the left side, the most pronounced decrease was measured in CA4 (relative change of volume in respect to the volume of the specific subfield in the DV− group with the sign of the number indicating either decrease or increase; −0.30), followed by CA3 (−0.27) and GC‐DG (−0.26) subfields. On the right side was the greatest decrease found in the CA3 subfield (−0.29), then CA4 (−0.27) and GC‐DG (−0.25) subfields.

### 3.3 GMV change in the Déjà vu

There was no significant change of GMV found in the DV+ group in comparison to DV− group in the present analysis. Nevertheless, there was a trend showing the most pronounced decrease in the fimbria, CA4, CA3, and fissure subfields. On the left side, the greatest extent of the relative decrease was found in the HATA (−0.04), followed by CA4, CA3, and fissure (−0.03). On the right side, the largest decrease was found in the CA3 and CA4 subfields (−0.03), followed by GC‐DG (−0.02).

In some of the subfields, there was even an insignificant increase in the SCV found—on the left side it was fimbria (0.04) and tail (0.01). On the right side, it was HATA (0.05) and fimbria, tail and subiculum (0.01).

### 3.4 GMV change in Schizophrenia

There was no significant change in the GMV comparing SCH vs DV− groups. Anyway, the trend was on the left side, the greatest decrease was found in HATA (−0.11), subiculum (−0.05), CA4 and CA3 (−0.04).

In the right sided hippocampus, the greatest extent of the decrease was found in the presubiculum (−0.04), CA3 (−0.03), and HATA (−0.02) subfields.

The increase in the GMV was found in the bilateral tail (0.01) and fimbria (0.02) and right CA1 (0.02) subfields.

### 3.5 Spatial distribution of GMV decrease

**Figure 2:**
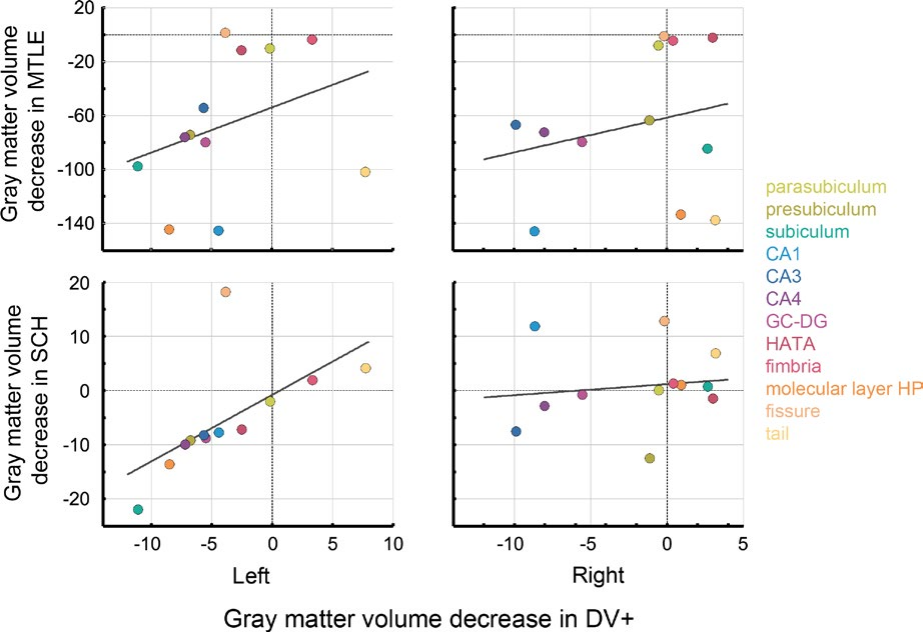
Scatterplots representing the correlation in gray matter volume (GMV) decrease relative to the DV− group (in mm^3^) between the DV+ and schizophrenia (SCH) or mesial temporal lobe epilepsy (MTLE) groups in the distinctive hippocampal subfields (colour coded) in the left/right sided hippocampus. The *x*‐axis value indicates GMV reduction for the DV+ relative to DV− group; the *y*‐axis indicates GMV reduction for SCH (left) or MTLE group (right) relative to DV−. Marks of the left SCH group are located at a shorter distance from the line of the best fit relative to those of the MTLE group, illustrating the higher correlation between DV+ and SCH compared to DV+ and MTLE at the left side

To assess the level of similarity, we compared correlation coefficients based on dmCSV and computed for DV+ vs SCH and DV+ vs MTLE. The correlation coefficient reached statistical significance for the left hippocampus for SCH vs DV+. Otherwise were the correlation coefficients without statistical significance. The values of the correlation coefficients are listed in Table 1 and the trend of the correlation coefficients across subfields is illustrated in Figure 2.

**Table 1:** The similarities of patterns of gray matter volume (GMV) changes among subgroups based on correlation coefficient

	Left hippocampus		Right hippocampus	
	SCH	MTLE L	SCH	MTLE R
*DV +*	**.637 (.013)**	.337 (.142)	.135 (.338)	.228 (.238)

MTLE, mesial temporal lobe epilepsy; SCH, schizophrenia.

The table shows correlation coefficients and *p*‐values (in brackets). Statistically significant result marked bold.

Our previous study (Brázdil et al., 2012) with healthy individuals showed a morphological correlate of nonpathological DV in terms of GMV decrease throughout a pattern of subcortical structures encompassing the hippocampi. In these regions, GMV was correlated inversely with the frequency of DV experience. On this basis we decided to examine differences in GMV between the distinctive hippocampal subfields according to the frequency of DV experience in the DV+ group. No statistically significant correlation between the GMV and the frequency of the DV has been found.

## 4 DISCUSSION

To the best of our knowledge, this is the first study that examines the morphological correlates of DV at the level of hippocampal subfields. This level of analysis did not reveal statistically significant change in GMV considering specific hippocampal subfields.

On the other hand, a significant change in the GMV of distinctive hippocampal subfields was found in the MTLE group relative to DV− in all hippocampal subfields with the exception of bilateral HATA, fimbria and fissure subfields. There was no significant change in hippocampal GMV found in DV+ or SCH groups.

The expected change of the GMV in conditions such as déjà vu and schizophrenia is subtle considering their nature and in the large number of recognized subfields the change is spread finally losing statistical significance. On the other hand, expected decrease in the case of MTLE with hippocampal sclerosis is much more prominent, which may be the cause it was the only difference spotted.

Concerning the spatial distribution of GMV decrease, we found statistically significant correlation for the left hippocampus for SCH vs DV+. Otherwise there was no statistically significant correlation. Thus, contrary to our hypothesis, GMV reductions in healthy individuals experiencing DV resembled more closely the pattern of GMV reduction observed in SCH patients.

Considering SCH group, no significant change has been found across hippocampal subfields which corresponds to some histological studies (Walker et al., 2002). Anyway the insignificant trend showing most profound decrease in the HATA, presubiculum, subiculum, CA4 and CA3 subfields correspond to some degree with the recent study of Haukvik et al. (2015) with the exception of HATA subfield, which was not recognized in the study. Mathew et al. (2014), however, found the most prominent decrease in CA4‐DG, subiculum and CA2‐3. This discrepancy could be explained by the work of Kühn et al. (2012), who report a negative correlation between positive symptoms expression and the volume of bilateral subfields CA2‐3 and CA1. Unfortunately, Mathew et al. do not specify the PANSS scores recorded from their subjects, thus a comparison with our SCH group cannot be made. CA1 sparing in the context of SCH is consistent with postmortem studies (Harrison, 2004).

For the MTLE group, our finding of GMV decrease in the majority of the hippocampal subfields is consistent with recent studies (Kim, Suh, & Kim, 2015; Schoene‐Bake et al., 2014). The specific pattern of GMV reduction also differed slightly, as Kim et al. measured the absolute decrease and found the most extensive reduction in ipsilateral CA2‐3 followed by subiculum and CA4‐DG. Our study on the other hand measured relative decrease considering volume of the particular subfield and found the maximal relative decrease in ipsilateral CA4 followed by CA3 and GC‐DG. Sparing of the HATA, fissure and fimbria subfields has not yet been observed in the literature considering the fact, that these subfields were not recognized.

Hippocampal sclerosis present in all MTLE subjects brings certain difficulty to the evaluation of GMV decrease, considering the ongoing discussion whether it is in fact a cause of epilepsy, or rather a consequence of seizure activity itself (early febrile seizures, episodes of status epilepticus or repetitive brief seizures; Thom, Zhou, Martinian, & Sisodiya, 2005). On the other hand, the hippocampal sclerosis is present in approximately 70% of “non‐lesional” MTLE (Cascino et al., 1991) and provides certain confidence to MTLE diagnostics.

The similarity of nonpathological DV to an ictal event was reported for the first time by Wilder Penfield more than sixty years ago (Penfield, 1955). Such qualitative similarity of DV experience in pathological and nonpathological instances was recently confirmed (Warren‐Gash & Zeman, 2014), as were similarities in neuroanatomical correlates (Brázdil et al., 2012) suggesting a common underlying process. Altered neural signaling within memory‐related medial temporal brain structures resulting in changes in GMV in covariating structures is supposed to underlie both pathological and nonpathological DV (Shaw et al., 2015).

Our primary expectation was therefore to find a greater similarity of GMV reduction pattern between the DV+ and MTLE groups compared with the DV+ and SCH groups. Ultimately, however, our results displayed a greater resemblance between the pattern of GMV pattern in the DV+ and SCH groups. We do not consider such resemblance to reflect a direct link between the two conditions. Rather, we hypothesize that this represents an unequal vulnerability of the distinctive subfields to these conditions.

The hippocampus is an especially plastic and vulnerable region of the brain (McEwen, 1999). This vulnerability was linked to a variety of insults including seizures (Cavazos, Das, & Sutula, 1994; Kotloski, Lynch, Lauersdorf, & Sutula, 2002; Mathern et al., 1996; Tasch et al., 1999), ischemia (Byeon et al., 2015) or inflammation (Malaeb et al., 2014; Marsland et al., 2015; O’Donovan et al., 2015), as well as gene‐environment interactions and physiological influences such as early life psychosocial stress (Butterworth, Cherbuin, Sachdev, & Anstey, 2012; Lindauer et al., 2005; Teicher, Anderson, & Polcari, 2012; Wang et al., 2010) or sleep deprivation (Novati, Hulshof, Koolhaas, Lucassen, & Meerlo, 2011). These factors, especially when they occur early in development, have been linked to hippocampal atrophy, alterations of postnatal neurogenesis and neuronal hyperexcitability (McEwen, 1999; Novati et al., 2011).

The mechanism of a deleterious stress action consists of a prolonged glucocorticoid (GC) exposure. The actions of these adrenal steroids occur predominantly in the hippocampus, a structure particularly rich in glucocorticoid receptors. This, in turn, leads to GC‐induced defense mechanism reduction, neuronal atrophy, or even neurotoxicity (Sapolsky, 1996).

Specifically, the CA3 subfield shows an abundance of GC receptors that allow for chronic restraint and psychosocial stress to produce atrophy of its pyramidal neurons apical dendrites (McEwen, 1999) and depletion and reorganization of synaptic vesicles in CA3 mossy fiber terminals (Magariños, Verdugo, & McEwen, 1997). Chronic elevations in corticosterone have also been shown to produce a suppression of synaptic plasticity in the dentate gyrus (Pavlides, Watanabe, & McEwen, 1993). Evidence of the deleterious effect of stress exposure on selected subfields can be found in the case of childhood maltreatment, where early exposure to elevated GC levels is believed to suppress neurogenesis and advance to GMV decrease in CA3, CA4, GC‐DG, and subiculum, respectively (Teicher et al., 2012).

Another condition indicative of a detrimental effect of excessive stress exposure is PTSD. Sufferers of PTSD have been found to express selective GMV loss in the CA3 and GC‐DG subfields, together with suppression of neurogenesis and dendritic branching in these structures (Wang et al., 2010).

Considering our results with most pronounced GMV decrease in corresponding subfields in all studied groups together with existing findings of specific hippocampal plasticity we hypothesize, that various adverse conditions cause GMV decrease in specific increasingly vulnerable subfields in the course of neurogenesis. Déjà vu, schizophrenia, and MTLE (together with PTSD and childhood maltreatment) then represent various magnitude of consequent aberrant functioning depending on the level of hippocampal damage.

Thus, we conclude that the HATA, CA3, CA4, GC‐DG, and subiculum subfields of the hippocampus are likely to be more vulnerable to adverse conditions. This conclusion is supported by our finding of the most pronounced GMV decrease in these particular subfields in all studied groups, with the exception of the right hippocampus in which presubicular GMV decrease was most prominent for patients with schizophrenia. This assumption converges nicely with the studies of childhood maltreatment and PTSD mentioned above, with the exception of HATA which was not recognized in previous studies.

Certain limitations of our study demand attention. Although the resolution of raw MR images of approximately 1 mm isotropic is considered as high spatial resolution in neuroimaging, still it is some limitation of our study as it is quite near the transversal size of some parts of hippocampus. Thus, the results might be slightly biased toward false negative results by the imperfect accuracy of segmentation process which may occur in some subjects (Kim, Mansi, Bernasconi, & Bernasconi, 2012). Another limitation consists in comparing clinical groups that differ in terms of “disease” duration and severity. Moreover, considering its unpredictable nature, the only possibility to study déjà vu is in between the events. Together with the fact, that it is a phenomenon based on the patient self‐report it brings a certain difficulty in proper assessment both of the frequency and experience itself. Imbalance in the age and gender distribution among groups might also potentially bias our results. We have, however, regressed the potential effects of these nuisance parameters out of the data, a well‐established method in neuroscience. Additionally, the effects of laterality and medications were difficult to interpret in this context. There are studies reporting no significant effect of antipsychotic medication on hippocampal subfields volume (Brambilla et al., 2013; Kühn et al., 2012; Wang et al., 2008; Zierhut et al., 2013). On the other hand, certain studies report some level of sensitivity to antipsychotic medication in particular subfields (Mamah et al., 2012; Panenka et al., 2007). Analyses of larger patient groups are needed in order to provide a detailed characterization of volumetric change, and to control for the potential effects of modifying factors. Future studies should also compare GMV within hippocampal subfields in other disorders, such as depression, insomnia or Alzheimer’s disease.

## CONFLICT OF INTEREST

None declared.
